# Domestic Summary

**Published:** 1840-04-04

**Authors:** 


### DOMESTIC SUMMARY.

*University of Pennsylvania.*—During the past session, four hundred and forty-four students were matriculated at this institution. At the late commencement, one hundred and fifty-nine graduates received the degree of doctor of medicine, making, with four at the previous July, a total of one hundred and sixty-three for the current year.

*Medical Department of Pennsylvania College.* We learn that eighty-seven students attended the recent opening session of this institution, and that degrees were conferred upon twenty-seven graduates.

*Albany Medical College.*—In this institution, the department of Materia Medica and Pharmacy has lately been changed to Materia Medica and Natural History, and the department of Chemistry and Natural History has been changed to Chemistry and Pharmacy.

Professor D. M. McLachlan has been elected Professor of Obstetrics and Diseases of Women and Children, in place of Professor Bedford, resigned.

Professor E. Emmons has been elected Professor of Materia Medica and Natural History, in place of Professor McLachlan.

And Lewis C. Beck, M. D., of Rutger's College, has been elected Professor of Chemistry and Pharmacy, in place of Professor E. Emmons.

*Report of the Obstetric Department of the Philadelphia Dispensary, for First, Second and Third months.* JOSEPH WARRINGTON, M. D., Accoucheur.

Forty women have been delivered of 43 children, at or near the full term of utero-gestation—three women having twins.

Twenty-four of the children were boys, 19 were girls. In two of the twin cases, the children were boys, in the other case they were girls.

The average duration of labour in 10 cases, was thirteen hours twenty-four minutes—the extremes being two, and thirty-three hours.

The average time occupied in the spontaneous delivery of the placenta in 16 cases, was nineteen minutes, the extremes being five and seventy-five minutes.

In two cases, the placenta was withdrawn from the os uteri and vagina after it had occupied that situation, in one case one hundred and twenty, and in the other two hundred and forty minutes, being in these instances retained merely for want of expulsive effort.

In another case, the placenta was extensively studded with calcareous deposites, without being accompanied with any feebleness or indisposition of the child.

Of 37 cases of cephalic presentation, 14 were in the first, one in the second, two in the third, one in the fourth, and one in the fifth position of the vertex; no other variety was noted.

The case which presented the occiput in the fifth position, was converted into the first by acting upon the right temple with the finger.

The forceps were applied at the superior strait in one of the cases of third position, and the patient safely delivered, after a tedious and exhausting labour.

In the other case of third position, the delivery was facilitated by converting it into a first position.

In the case of adhesion of the edge of the placenta and a portion of the membranes, the hæmorrhage, after delivery, became alarmingly profuse. Ergot, suspended in brandy, was administered, and as soon as the adhesions were broken up, the complete contraction of the uterus was aided by friction, followed by a graduated compress upon the hypogastric region, and a tight bandage around the lower part of the abdomen.

In one case, severe and extensive peritonitis came on the third day after delivery; this was treated by copious and repeated bleeding, general and local, calomel, Dover's powder, castor oil, and, towards the conclusion, free vesication over the abdomen.

One patient suffered from severe uterine engorgement, with exalted sensibility, which was speedily relieved by free venesection and saline cathartics.

All the women recovered.

One child had hare-lip, which was successfully operated upon at about five weeks old, by Dr. Mütter.

One male child suffered from sabulous deposites in its urethra, and difficulty in urinating. One female child had retention of urine; both required the use of the catheter for several days after birth; they recovered, and all the other children, except one, which died in convulsions on the fourth day after delivery, have done well.

Nine pupils have participated in the "practical instructions," and attendance upon cases conducted by the accoucheur, during the period embraced in the above report.

---

*Extract from the Report of the Surgeon-General of the United States.*

"The number of cases of indisposition which have been under treatment by the medical officers of the army, and private physicians, temporarily in the service of the United States, during the last twelve months, was 22,849; 22,248 of which occurred within the past year; 649 being cases that remained of the preceding year. Of the whole number of persons reported sick, 21,940 have been restored to duty, 131 have been discharged the service, 55 have deserted, and 214 have died; leaving, on the 30th of September, 1839, 909 still on the sick report.

From the monthly returns and other reports it is estimated that the aggregate mean strength of the army for the last year, was 8,950; and as the number taken sick during the year, was 22,248, and the aggregate of deaths was 214, it will appear that the proportion of cases of disease to the number of men in service, was as 2½ to 1, or 249 per cent.; the ratio of deaths to the number of men as 1 to 42, or 2 4-10ths per cent.: and the proportion of deaths to the number of cases treated as 1 to 107, or a fraction less than 1 per cent.

From our observations, it is found that the troops which have taken the field from southern stations, have suffered less from sickness, and lost fewer men by disease, since they came into Florida, than while they were stationary at their posts. Nor have the corps from the north suffered from disease and death to the extent that is generally believed.

The reports in this office, from every section of the United States, show this result; and as I am satisfied of the fact, I deem it to be my duty to correct an error of opinion that seems to pervade the country to the manifest injury of the military service.

In further illustration of this subject, the statements marked C and D, are submitted.

The law requiring an examination of candidates for appointment and of assistant surgeons for promotion in the medical department of the army, has been rigidly enforced.

Two junior surgeons whose examination for promotion had been unavoidably deferred, and three assistant surgeons of five years standing, were ordered to present themselves, and thirty-six applicants for appointment to the medical staff of the army, were invited to appear before the medical board lately in session, at New York. The surgeons and assistant surgeons having undergone a thorough examination upon all the branches of medical science, and received a favourable report from the board, the first two were sustained in their advanced position, and the last three rendered *legally* qualified for promotion. With the candidates for admission into the army, however, the result of the examination was very different. Of the thirty-six who were invited to appear before the board, twelve declined the examination, (two, after having reported to the board,) two were excluded on account of their age, and twenty-two were examined; and of these last, five only were found to possess all the qualifications essential to an appointment.

It may be that we have erected too high a standard of merit—that too much is exacted from the human intellect; we are not conscious, however, that more has been asked, than ordinary talents, a good primary education, and the actual study of the science of medicine, can attain. At all events, some few have reached the highest scale of excellence; and while as many of these choice spirits can thus be secured. as will fill our ranks in each succeeding year, we shall not relax in our requirements upon those who claim to be admitted into the medical staff of the army.

But to account for the humiliating result of the examination on the present and on the former occasions, we have only to look to the system of education which now obtains in the country.

The facilities of acquiring medical knowledge, or rather of becoming professional men, are so great, that many persons are seduced into an attempt to become physicians, without the basis of an education. There are others again, who having received a good primary education, and also passed through a regular classical or collegiate course, (and thereby rendered qualified for scientific pursuits,) are induced from motives of economy and convenience, or with the view of sustaining institutions of their own state, to enter some of the small medical schools, where they cannot possibly have the advantages of anatomical dissection, (the ground work of the profession,) or the means of clinical instructions upon an extended scale. A knowledge of the science of medicine is not, like divinity and law, to be acquired by reading books in the closet, and listening to the reading of a course or two of lectures; it can only be attained by seeing and feeling, in connection with the knowledge acquired from books.

The great multiplication of medical schools in every section of the country, together with the proverbial facilities of becoming licensed practitioners, has so lowered the standard of professional excellence, and so manifestly degraded the medical character of the United States, that the present system will be, it is to be hoped, by a more enlightened public opinion ere long put down. The interests of the country is so much divided by these various institutions, and the patronage to each is consequently so small, that many of our ablest medical men will not accept places in them; were it practicable, however, for the professors to obtain adequate compensation for their services, it would be impossible to find professional men enough of talents and attainments to occupy the several chairs in the innumerable medical schools in every town, village, and cross-road place, throughout our states and territories.

Every officer of this department has been almost continuously on duty, seldom more than two at a time on leave of absence, and in no instance more than four even temporarily absent from duty. The duty in Florida, particularly, has been so irksome as to produce some murmuring from two or three individuals of the corps; but as those persons, generally, grumble the most who have the least cause of complaint, and are least deserving, perhaps, of special consideration, I have not permitted these symptoms of dissatisfaction to interfere with the general arrangements of the department, or to paralyze, for a moment, our efforts to meet the actual requirements of the service.

The service in Florida, to most of the medical officers employed there, has been, indeed, not only irksome but exceedingly laborious and hazardous, many of them having, from the very dispersed state of the troops, to give their attendance to two, three, or more posts and stands; frequently passing from one station to another without an escort, and occasionally under the fire of the enemy.

Among others whose lot it was to perform more than ordinary duty, was the accomplished surgeon, Richard Clark, who, in the height of his usefulness, was lately cut off by disease.

Doctor Clark having been called to a distant post, where the whole command, officers and men, lay prostrate from disease, he at once gave all the energies of his mind and body to the assistance of his suffering comrades; and while thus engaged in administering, by day and by night, to the diseases and to the wants of the sick, he was inhaling the noxious vapours of the place, even to his own destruction. After rendering much assistance, and, indeed, all the aid practicable, he himself sank to the ground, and in a day or two afterward yielded up his gallant spirit, a martyr to the calls of humanity and his country's good.

For this very severe and perilous duty—this extraordinary devotion to their country's cause, (this extra service being peculiar to themselves, and not absolutely to be required of them,) these officers are entitled to a full measure of praise; and I do not hesitate thus to express the high sense that I entertain of their public services and of their public worth.

In obedience to your instructions, a commission consisting of three medical officers was appointed to examine the banks of the Ohio river, between Pittsburg and Wheeling, and also the shores of Lake Erie, with the view to the selection of sites for marine hospitals. The board having completed the reconnoisance of the country designated, and collected a mass of useful information touching the subject of inquiry, made a detailed report of its proceedings, recommending a change of location for the hospital on the upper Ohio, from Wheeling to the vicinity of Pittsburg, while it sustains the decision of the former board in favour of Cleveland, over Presque Isle, and other points, as the site for the hospital on the shore of Lake Erie. As this report takes a different view of several matters and circumstances from that of the former commission, I beg leave to recommend them both to your attentive perusal and consideration.

From these two able reports and the various statistical and other documents accompanying them, the president will receive all the lights upon the subject of inquiry of which it is susceptible, and can consequently determine understandingly upon the points at which the contemplated hospital shall be erected.

I have, within the past year, with the assistance of one or two medical officers, brought up almost all the back business, which had for years, in consequence of the want of force in the office, been accumulationg upon the department.

The accounts of every officer of the department, and of private physicians in the service of the United States, so far as this office is concerned, have been brought up and closed to the 31st of March; and, with the exception of four or five, whose returns have not come in, even to the 30th of September of the present year.

I have also had prepared, or have now in a state of forwardness, a meteorological register, embracing thermometrical observations, for a series of years, in every section of our States and Territories; also, a report on the vital statistics of the army and the medico-topography of the military stations, extending over a period of twenty years; all which will be ready for the press in a few days, should their publication be authorized by Congress or by the Department of War.

I beg leave, in conclusion, to call your attention again to the fact that the services of another clerk are indispensable to this office. While I have the control of the department I shall keep the business up; and if I cannot obtain the aid of a regular clerk, I must call into requisition the occasional services of one or more of the medical officers of the army.

All which is respectfully submitted.

TH. LAWSON, Surgeon General.

Hon. Joel R. Poinsett, Secretary of War, Washington."

The report of the Surgeon-General is interesting from the valuable statistical matter which it contains, and we are glad that the numerous documents which have been collected by the industry of the army surgeons, are to be made the basis of further publications from the office at Washington.

The rigorous examinations which are now required from all applicants for admission into the army and navy, will undoubtedly exercise a wholesome and efficient influence in stimulating the candidates to become thoroughly conversant with their profession, and if conducted upon a liberal, though rigid footing, may have an indirect effect in elevating the standard of medical education and increasing the requisites for graduation now required by our medical colleges. In this respect, therefore, we, as civilians, are interested in the rigorous examinations of candidates for admission both into the army and navy. The peculiar circumstances in which the military and naval boards are placed enable them to require these conditions. The army and navy need but a small number of assistant surgeons; the candidates are always much more numerous than the places to be filled; hence it is not only feasible, but most expedient, to insist upon a maximum degree of knowledge, and to reject all who do not come up to this level. In medical colleges the case is widely different; they are obliged, for many reasons, to grant diplomas to all who do not fall below mediocrity. The one class is properly enough supposed to be composed of those who can pass an examination much above the average, the others of those who do not fall much below it.

It is perfectly well understood that the requisitions of all medical colleges might be made more severe, and that a longer period of study, and a more rigorous practical examination would ensure a higher average of qualifications. Whether all the medical schools in the United States would choose to exact these qualifications, we do not know, but we are perfectly aware that some of them at least would be more than willing to enforce a higher standard of medical knowledge, and that as far as these are concerned, the difficulty lies not with the teachers, but with the pupils. It would be absurd to require a course of study which is incompatible with the eager desire of young men to enter upon an active career, and would, therefore, if adopted by one institution, induce the great mass of students to resort to schools where a modicum of knowledge could be obtained at an easier rate, and the ultra rigid schools would soon find themselves without a class, unless they could offer advantages sufficient to induce the pupils to prolong their stay and to extend their circle of studies. These advantages are presented by the army and navy to those who have a taste for the career of military or naval surgeons. A successful candidate obtains, at once, an honourable position, and a comfortable, if a restricted income; he is willing, therefore, to undergo the risks of an examination, knowing that if he succeeds, he will gain many important advantages. Of these candidates for the army, it seems but a small proportion was successful, that is, less than a fourth of them who finally presented themselves for examination, or five in twenty-two. At the last examination for the admission of assistant surgeons into the navy, we understand the proportion of successful candidates was more considerable, twelve in twenty-six.

Both these examinations are regarded as severe, and as justly severe, yet we see that there is no proportion between the number of successful candidates for the two places. The explanation of this is easy; the applicants for the navy, as a general rule, look forward to the situation from an early period of their studies, and prepare themselves expressly for the examination. This is not, we believe, so frequently the case with applicants for the army. We have known several candidates who have prepared themselves for the examination, and have passed it without difficulty; but it is a matter perfectly well understood, that a number of permits are obtained by men either actually engaged in practice, or who have unsuccessfully attempted to obtain civil practice. A physician who has been long engaged in the pursuit of his profession, is rarely able to pass an examination upon elementary details necessary to be learned, but not necessary to be retained. An ordeal of this kind, would, we fear, find many of the army surgeons as much embarrassed as it would other practitioners of equal attainments and skill. An examination requires special preparation, and no one, who is accustomed to a test of this kind, would trust to his own qualifications, unless he had brushed up his knowledge and returned to his elementary studies.

Our efforts, like those of many of our contemporaries, are directed to the upholding of a high standard of medical education. We are neither tempted by interest nor by inclination to depress the scale; and we do not think that the medical schools which require a strict examination are the less attended. On the contrary, we know that the school which is supposed by students to be amongst the most rigorous, is by far the largest in the United States. We even believe that the qualifications for a degree, and the number of branches taught, might be increased to the advantage of the school, and to the great benefit of the pupils, but we know that this increase of requisites must be gradual, and adapted to the habits of our youth, and the wants of the country. We have no doubt that some means will be offered, which will, in time, ensure a higher standard of knowledge; whether these means will be derived from the combined action of professional men, or from a national medical society, must remain for the present unknown. We do not, however, think that the unsuccessful candidates for the army present a fair average of the attainments of our younger professional brethren. Those who have received the benefits of the most thorough and practical course of instruction, for the most part, prefer the chances of civil practice, which, although often unproductive, still offers the chance of a more lucrative, and, to most men, a more desirable situation. Those candidates whose attainments are of a higher order, succeed in their object, and we, as civil practitioners, can meet them in the confident belief, that the fact of their admission into the army or navy is in itself a proof of professional attainments. We object to the inference that has been drawn from the report of the Surgeon-General, but we are glad he is uniting his efforts to our own, in stimulating the students of medicine to look to a higher standard than that with which some of them are now contented. This is, however, a work of time, and can only be brought about by a judicious and gradual influence.

